# Exploration of different classes of metrics to characterize motor variability during repetitive symmetric and asymmetric lifting tasks

**DOI:** 10.1038/s41598-019-46297-3

**Published:** 2019-07-08

**Authors:** Alireza Sedighi, Maury A. Nussbaum

**Affiliations:** 1 Bone and Joint Center, Department of Orthopaedic Surgery, Henry Ford Health System, Detroit, Michigan 48202 USA; 2 Industrial and Systems Engineering, Virginia Tech, Blacksburg, 24061 USA

**Keywords:** Motor control, Dynamical systems

## Abstract

The substantial kinematic degrees-of-freedom available in human movement lead to inherent variations in a repetitive movement, or motor variability (MV). Growing evidence suggests that characterizing MV permits a better understanding of potential injury mechanisms. Several diverse methods, though, have been used to quantify MV, but limited evidence exists regarding the merits of these methods in the occupational context. In this work, we explored different classes of methods for characterizing MV during symmetric and asymmetric box lifting tasks. Kinematic MV of both the whole-body center-of-mass (COM) and the box were quantified, using metrics derived from a linear method (Standard Deviation), a non-linear method (Sample Entropy; an index of movement regularity), and a novel application of an equifinality method (Goal Equivalent Manifold; an index related to the set of effective motor solutions). Our results suggest that individuals manipulate regularity and the set of effective motor solutions to overcome unwanted motor noises related to the COM. These results, together with earlier evidence, imply that individuals may prioritize stability over variability with increasing task difficulty. Task performance also appeared to deteriorate with decreasing variability and regularity of the COM. We conclude that diverse metrics of MV may be complimentary to reveal differences in MV.

## Introduction

The human body has substantial kinematic degrees-of-freedom that can be used to perform specific tasks, and a challenging question is how the central nervous system (CNS) can overcome such kinematic redundancy^1^. Bernstein^2^ noted that reproducing a specific movement is impossible, and that there are inherent variations in movements because of the many available solutions for executing a task. These variations have been treated as an essential characteristic of motion^3^ and termed motor variability (MV). MV is now often considered as a regulator of motion^4^ to increase flexibility and adaptability^5^; therefore, Latash^6^ termed these variations the “principle of abundance” rather than a “problem of redundancy”. There may be an optimal amount of MV^7^ – neither too much variability nor movements that are too rigid – and some evidence even suggests that MV has a key role in preventing musculoskeletal disorders^8–12^ and improving performance^8,13^.

Earlier reports have suggested an association between the amount of MV and musculoskeletal pain. As examples, both chronic neck/shoulder pain^14^ and low back pain^15^ were found to be associated with less movement variations. Further, pain-free individuals increased MV after fatigue, yet individuals with low back pain did not use this movement strategy to adapt with fatigue^16^. Such evidence suggests that individuals may avoid painful movement solutions and that this leads to a reduction in MV^17^. Some biomechanists have also hypothesized that increased MV may be beneficial, in terms of preventing injury and pain, suggesting that more variations in a repetitive task decrease mechanical loads on specific soft tissues, and that consequently this mechanism may prevent the development of pain^9,17^. In support of this hypothesis, individuals who developed pain had a lower amount of trunk MV compared to a group without pain^8^. In summary, existing evidence does appear to support a key role for MV in the development of musculoskeletal pain and injuries.

There is also evidence for an association between MV and task performance. Individuals can improve or preserve task performance by utilizing more movement solutions when performing a given task^13,17^. In the occupational domain, Mirka and Marras^18^ found high variations in muscle activity (execution variables) during a repetitive lifting task, but these variations changed such that the generated external torque (performance) stayed fairly consistent. Gates and Dingwell^19^ found that when participants became fatigued in a repetitive sawing task, their movement pattern changed to maintain goal performance on the task. It is worth noting that, for skilled tasks such as gait, individuals may increase MV to overcome unwanted perturbations and to preserve performance. In less skilled tasks, however, individuals may explore more motor solutions to improve performance. Thus, more variation in execution variables might be beneficial to preserve or improve task performance.

Repetitive lifting is a common occupational task and is considered as an important cause of more than half of occupational musculoskeletal disorders (MSD)^20^, especially low back pain^21^. Asymmetric lifting conditions in particular increase the risk of MSDs^22–24^. Previous studies have also found that task symmetry affects motor control strategies (e.g., stability and movement variability)^25–27^. Given the evidence that MV might be associated with task performance and injury risks, we suggest that quantifying the extent of MV in the context of repetitive lifting may identify new approaches to evaluate MSD risk factors (e.g., task symmetric) and performance related to this important occupational task.

Characterizing MV remains as an important challenge, however, arising from the fact that there are diverse methods to quantify MV. These methods exist in three distinct *classes*^28^. Traditional methods (linear class of methods) form the first class, and are based on descriptive statistics. The second class stems from chaos theory (nonlinear class of methods), with several tools presented recently in the field of human movement (see Stergiou^28^ for an overview). The third class of methods is based on the abundant degrees-of-freedom available to perform an action, which Cusumano and Cesari^29^ termed “equifinality”. There are several methods to quantify MV based on equifinality, including the uncontrolled manifold (UCM)^1,30^, tolerance-noise-covariation^31^, the minimum intervention principle^32^, and the Goal Equivalent Manifold (GEM)^29^. Among these, GEM is the only method from the equifinality class that can simultaneously quantify the magnitude and temporal structure of variability^33,34^.

Each class of methods provides different information regarding MV, and a rationale is needed to select a specific one^17^. However, little evidence exists regarding the benefits/limitations of these classes of methods for characterizing MV^17^, particularly in the context of repetitive lifting. This gap hinders our ability to select an appropriate class of methods for addressing a specific research question related to MV. Other challenges are that previous studies have used different kinematic parameters to characterize MV^17^. However, there are presently no guidelines for selecting a kinematic parameter that is likely controlled by the CNS and that specifically reflects the kinematic control strategies in terms of regularity, flexibility, and the magnitude of variability. Thus, there are open questions about quantifying MV in the occupational domain. Here, we first aimed to explore which kinematic parameters might be controlled by the CNS in the context of a common occupational task (lifting/lowering). Our second aim was to compare and contrast the use of the different classes of methods for characterizing MV related to task symmetry (one important injury risk factor). Regarding the second aim, we first hypothesized that outcomes reflecting variability would differ between task symmetry conditions. Secondly, we hypothesized that metrics obtained from these diverse classes of method would have differing sensitivities to task symmetry conditions.

## Methods

### Participants and procedures

The current work was a secondary analysis of data obtained in a prior study^27,35^. Complete details are available in the cited reports, and as such the study is only summarized here. The Virginia Tech Institutional Review Board (IRB) reviewed and approved all of the experimental protocols, and all experimental methods were completed in accordance with relevant guidelines and regulations. Twelve participants were involved in the study (10 males and two females), and each provided informed consent prior to beginning the study. Participants performed 40 repetitions of lifting/lowering a box from/to knee/elbow heights. This was done both symmetrically, in the sagittal plane, and asymmetrically, to a shelf positioned 60° to the right side of the participant from the sagittal plane. Two shelves were included as the pick-up and put-down locations of the box, the heights of which were set such that the top of the box was at standing knee and elbow heights. Boxes were set to 10% of individual body mass, and lifting/lowering rate was controlled at 20 cycles per minute with a metronome; these task parameters were determined in pilot work to lead to minimal fatigue development. Participants were asked to grasp the box continuously and to use a free-style lifting technique but without moving their feet. Segmental kinematics and the box trajectory were tracked at 100 Hz using reflective markers. Raw data were low-pass filtered (bi-directional, 2^nd^-order Butterworth) with a cut-off frequency of 5 Hz. The initiation of each lifting cycle (lifting/lowering) was defined at the time when BOX velocity exceeded 3% of its peak value in that cycle subsequent to the time that the BOX was at rest on a shelf^36,37^. Data from first and last cycles were excluded from further analysis.

### Data analysis

To quantify MV in the lifting/lowering task, several kinematic parameters could be considered. In reaching tasks, for example, variations in movement patterns of end effectors have usually been investigated^37–39^. Here, performance of the end effector was evaluated by analyzing the BOX trajectory. It was also of interest whether the CNS might employ different strategies to control the end effector vs. body movement. MV of the whole-body center-of-mass (COM) was used to quantify the latter, since earlier results suggested that the CNS controls the COM to regulate movement^4,36^.

As noted earlier, there are three classes of methods for quantifying MV. One representative method was chosen from each class here, and metrics of MV obtained using these methods were compared to help understand which of the class of methods might better identify differences between the lifting conditions. Cycle-to-cycle standard deviation (SD, from the linear class of methods) and sample entropy (SaEn, from the nonlinear class of methods) were used, each of which has been applied to quantify MV in pipetting tasks^37,38^. In the context of MV, repetitive lifting and pipetting tasks appear similar, since in both the end effectors are considered to evaluate performance (i.e., the task involves repetitively moving a BOX vs. pipette at a constant rate between fixed origins and destinations). As discussed by Cusumano and Dingwell^33^, the GEM (from the equifinality class of method) may be the most appropriate method to study MV, and thus this method, using a new application, was employed to quantify trial-to-trial MV of the lifting task. These methods are explained in more details below.

Within the linear class of methods, we used cycle-to-cycle SD to quantify MV. Based on the work of Srinivasan *et al*.^37^, motor control strategies used to control the BOX and the COM can be evaluated by calculating the cycle-to-cycle SD of the mean speed (*V*) and path (*X*) of the BOX and COM, as well as the duration (*T*) of the lifting/lowering task. Here, *T* is the duration between the initiation of two consecutive cycles, *X* is resulting 3D trajectory of the BOX or the COM, and *V* = *X*/*T*. Similar to Lee and Nussbaum^35^, we developed a 3D linked-segment model (including 15 body links + the box) to calculate COM position. Thereafter, we quantified the magnitude of variability of the BOX and COM by measuring the cycle-to-cycle SD of the described variables (i.e., *V*, *X*, and *T*).

SaEn was used as a method in the nonlinear class to quantify MV. We applied a procedure similar to that developed by Richman and Moorman^40^ to compute SaEn of the increment of BOX and COM paths (see appendix A). SaEn is an index of regularity, which indicates the extent to which movement is predictable. Higher values for this index (i.e., SaEn) indicate that the signal is less repetitive (less predictable) and vice versa. As such, we assessed regularity of the BOX and COM kinematics, by computing SaEn of these two kinematic parameters. To improve the accuracy of the method, and to remove temporal correlated data points, we implemented a modified version of SaEn in which time delay^41^ and the Theiler window^42^ were incorporated in the definition.

**Figure 1:**
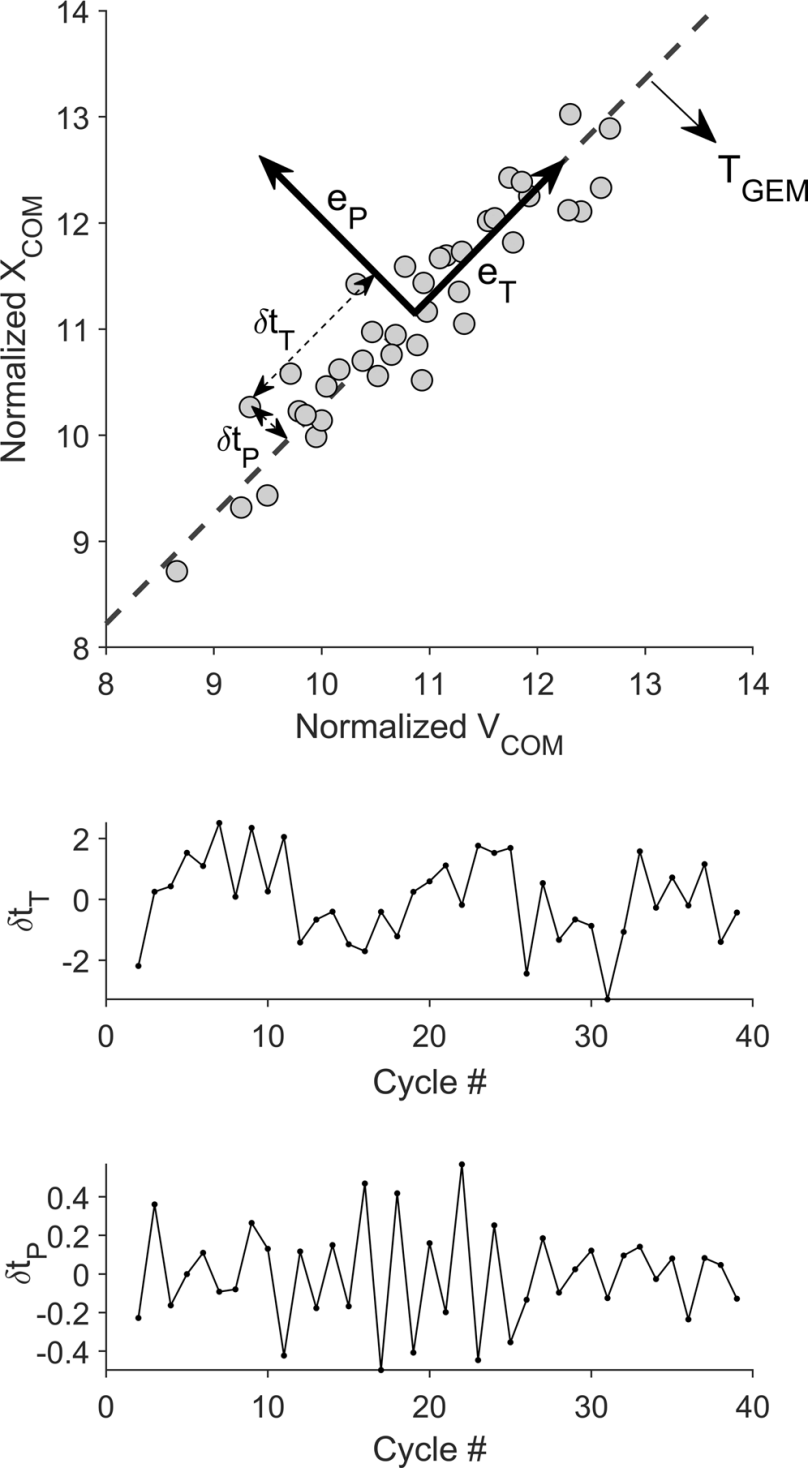
Representative example of movement variability for one participant based on the GEM method. Top: the distribution of X_COM_ and V_COM_ around the goal (i.e., constant time); Middle: deviations along the GEM direction (*δt*_*T*_) for each lifting/lowering cycle; Bottom: deviations in the non-GEM relevant direction (*δt*_*P*_) for each cycle.

To quantify MV using the GEM method within the equifinality class, we needed to define a main task goal. In the study from which the current data were obtained^35^, participants were required to maintain pacing of the lifting task; therefore, a constant time was considered as the main goal in our GEM analysis. In an earlier report^36^, we presented a time-GEM strategy method^39^ for a lifting task (see appendix B) and employed the same method here. As illustrated in Fig. 1, variability in each cycle can be computed in the GEM direction (*δt*_*T*_) and in the direction perpendicular to it (*δt*_*P*_), using:

1 $$[\begin{array}{c}\delta {t}_{T}\\ \delta {t}_{P}\end{array}]=\frac{1}{\sqrt{1+{T}_{GEM}}}[\begin{array}{cc}1 & {T}_{GEM}\\ -{T}_{GEM} & 1\end{array}][\begin{array}{c}{V}_{n}-{V}^{\ast }\\ {X}_{n}-{X}^{\ast }\end{array}]$$

where *X*_*n*_ and *V*_*n*_ were obtained by normalizing path and velocity of the COM or BOX to their respective SDs; *T*_*GEM*_ = *mean* (*X*_*n*_/*V*_*n*_); V^*^ is the mean of *V*_*n*_; and ${X}^{\ast }={T}_{GEM}\cdot {V}^{\ast }$.

To study the structure of variations, we computed the SD (*σ*) of both *δt*_*T*_ and *δt*_*P*_^33^. If σ(*δt*_*T*_) is substantial higher than σ(*δt*_*P*_), it indicates that participants maintained the GEM goal^33^. Similar to Decker *et al*.^43^, we calculated relative variability (σ(*δt*_*T*_)/σ(*δt*_*P*_)) to compare flexibility – the ability of individuals to use the set of effective motor solutions – between task conditions. A higher ratio indicates that the individual has more relative variability and vice versa^43^. Yet, these measurements (similar to UCM analysis) only reveal the average behavior of the system. To analyze the temporal structure of our time series data (i.e., *δt*_*T*_, *δt*_*P*_, and *δt*_*T*_/*δt*_*P*_), a Lag-1 autocorrelation method was used^39^. Several studies (e.g., Dingwell, *et al*.^39^) have suggested that two consecutive cycles are highly correlated, and thus this relationship can be expressed as:

2 $${S}_{i+1}=\lambda {S}_{i}+\xi $$

in which *S* and *ξ* are the time series and noise, respectively. Stability and (anti) persistence of the time series can be interpreted based on the *λ* value: *λ* > 0 → persistence; *λ* < 0 → anti-persistence; and *λ* = 0 → uncorrelated^39^. In the context of motor control, a higher value of *λ* suggests that the CNS has less control over the time series, and vice versa.

### Statistical analyses

In the original study^35^, half of the participants had experience in occupational lifting tasks and were regularly performing such tasks. To address potential influences on the results related to the level of experience, we completed preliminary analyses on the metrics obtained using the linear, nonlinear, and equifinality classes of methods. These preliminary analyses were done using mixed-factor analyses of variance (ANOVAs), including lifting symmetry (LS) and the level of experience. Results of these preliminary analysis indicated that effects of the level of experience were only statistically significant or substantial for *λ*(*δt*_*T*_) of COM. As such, the ANOVA model was maintained for analysis of *λ*(*δt*_*T*_) for COM. For all other outcomes, paired *t*-tests were used to assess the effects of LS. Parametric model assumptions were assessed, and we used a reciprocal square transformation of relative variability (i.e., *σ*(*δt*_*T*_)/*σ*(*δt*_*P*_)) to obtain normally-distributed model residuals. In all analyses, *p* values ≤ 0.05 were considered statistically significant. Where effects of LS were significant, summary results are presented as least-square means (LSM, with 95% confidence intervals). We used JMP (13.0.0, SAS Institute Inc., Cary, NC) for all statistical analysis. Further, the relative sensitivity of the different measures of MV (SD cycle-to-cycle, SaEn, and GEM method) to LS were assessed via their respective effect sizes (i.e., partial eta-squared, or ${\eta }_{p}^{2}$). The criteria provided by Cohen (1988) were used to interpret these effect sizes qualitatively: large if ${\eta }_{p}^{2}$ > 0.14, moderate if ${\eta }_{p}^{2}$ > 0.06, and small if ${\eta }_{p}^{2}$ > 0.01.

## Results

### Linear class of methods

**Figure 2:**
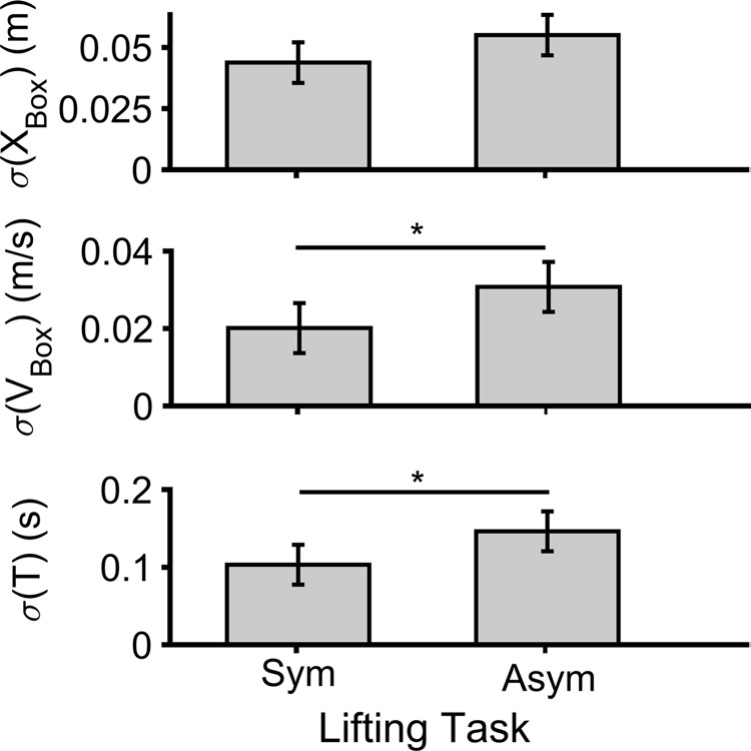
Cycle-to-cycle SD of the BOX and *T* for symmetric (Sym) and asymmetric (Asym) repetitive lifting/lowering (Top: variations of the BOX path; Middle: variation of the BOX velocity; Bottom: variations in the duration between two consecutive cycles). The symbol * indicates a significant difference between symmetry conditions (*p* < 0.05), and error bars indicate 95% confidence intervals.

Statistical results obtained using cycle-to-cycle SD are summarized in Table 1 and Fig. 2. We only observed significant effects of LS on cycle-to-cycle SD for the BOX, but not the COM. Cycle-to-cycle variability of the BOX path slightly increased from the symmetric to asymmetric conditions, though the difference only approached significance. A similar pattern of increased cycle-to-cycle variability with task asymmetry was also evident for the mean speed of the BOX and the duration of the lifting/lowering task, and in both cases these differences were statistically significant.

**Table 1:** Summary of statistical results (*t*-tests) for the effects of lifting symmetry on cycle to cycle SD (*σ*) of the whole-body center-of-mass (COM), BOX, and task duration (Time).

		*F*_(1,11)_	*p*	${{\boldsymbol{\eta }}}_{{\boldsymbol{p}}}^{2}$
*σ*(*T*)	Time	**6**.**787**	**0**.**024**	**0**.**382**
*σ*(*X*)	COM	0.435	0.523	0.038
	BOX	*4*.*461*	*0*.*058*	*0*.*288*
*σ*(*V*)	COM	2.038	0.181	0.156
	BOX	**6**.**577**	**0**.**026**	**0**.**374**

For the former two, results are presented for both mean speed (*V*) and path (*X*). For each, *F* values, *p* values, and effects sizes (${\eta }_{p}^{2}$) are provided. Bold fonts highlight significant effects, and italic font highlights effects that approached significance.

### Nonlinear class of methods

For SaEn, there was a significant effect of task condition only for the COM, but not the BOX (Table 2). SaEn for the COM was higher in the symmetric vs asymmetric conditions, with respective LSMs (CI) of 0.895 (0.864–1.026) and 0.737 (0.606–0.867).

**Table 2:** Summary of statistical results (*t*-tests) regarding the effects of lifting symmetry on SaEn of the whole-body center-of-mass (COM) and the BOX.

	*F*_(1,11)_	*p*	${{\boldsymbol{\eta }}}_{{\boldsymbol{p}}}^{2}$
SaEn(*x*_*COM*_)	**17**.**363**	**0**.**0016**	**0**.**612**
SaEn(*x*_*BOX*_)	1.401	0.261	0.113

For each, *F* values, *p* values, and effects sizes (${\eta }_{p}^{2}$) are provided. Bold fonts highlight significant effects.

### Equifinality class of methods

**Figure 3:**
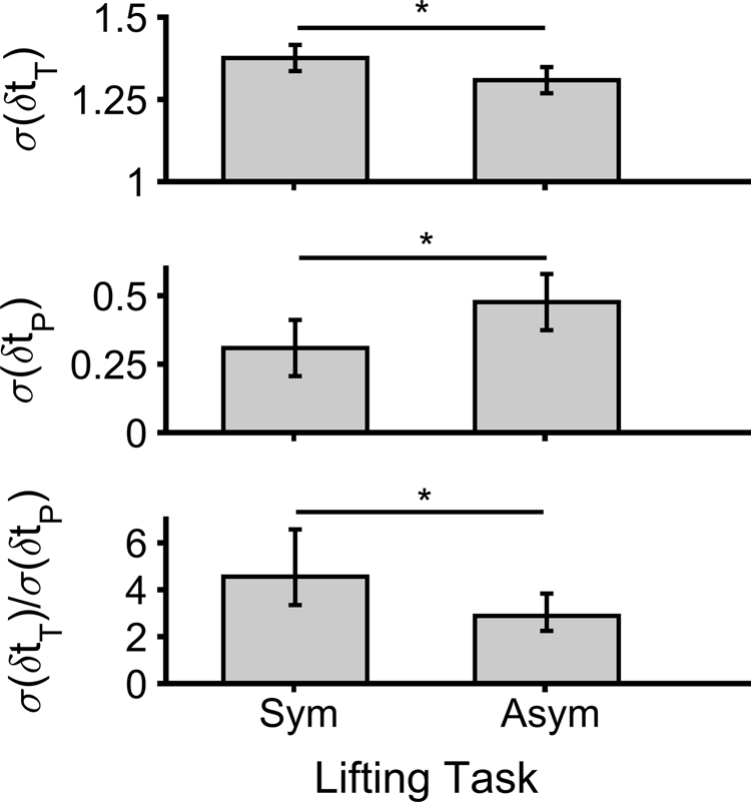
GEM result for the COM in symmetric (Sym) and asymmetric (Asym) repetitive lifting/lowering (Top: variability in the GEM direction; Middle: variability in the direction perpendicular to the GEM; Bottom: relative variability). The symbol * indicates a significant difference between symmetry conditions (*p* < 0.05), and error bars indicate 95% confidence intervals.

Effects of LS on metrics obtained using the GEM method are summarized in Table 3 and Fig. 3. The GEM method revealed significant main effects of LS on *σ*(*δt*_*T*_), *σ*(*δt*_*P*_), and *σ*(*δt*_*T*_)/*σ*(*δt*_*P*_) only for the COM, but not the BOX. For the COM, variability along the GEM (*σ*(*δt*_*T*_)), and relative variability (*σ*(*δt*_*T*_)/*σ*(*δt*_*P*_)), both decreased significantly from the symmetric to asymmetric conditions. However, the amount of variability in the non-relevant GEM direction (*σ*(*δt*_*T*_)) was higher in the asymmetric condition.

**Table 3:** Summary of statistical results regarding the effects of lifting symmetry on metrics obtained using the GEM method.

		*F*_(1,11)_	*p*	${{\boldsymbol{\eta }}}_{{\boldsymbol{p}}}^{2}$
*σ*(*δt*_*T*_)	COM	**6**.**981**	**0**.**023**	**0**.**388**
	BOX	3.541	0.087	0.243
*σ*(*δt*_*P*_)	COM	**6**.**492**	**0**.**027**	**0**.**371**
	BOX	3.291	0.097	0.230
*σ*(*δt*_*T*_)/*σ*(*δt*_*P*_)	COM	**5**.**722**	**0**.**036**	**0**.**342**
	BOX	2.635	0.133	0.193
*λ*(*δt*_*T*_)	COM	1.374	0.266	0.111
	BOX	<0.001	0.979	<0.001
*λ*(*δt*_*P*_)	COM	0.196	0.666	0.017
	BOX	0.214	0.653	0.019

For each, F values, *p* values, and effects sizes (${\eta }_{p}^{2}$) are provided, and bold fonts show significant effects. All results are from *t*-tests, except for *λ*(*δt*_*T*_), for which ANOVA was used (see Methods).

## Discussion

We found that individuals exhibited different strategies to control the BOX and the COM during repetitive lifting/lower tasks. Cycle-to-cycle variability of mean speed, BOX path, and cycle duration were all higher in the asymmetric vs. symmetric conditions. In contrast, there was lower regularity of the COM in the asymmetric task, as well as lower relative variability of this parameter (due to a lower variability in the GEM direction and a higher variability in the non-relevant GEM direction).

In this exploratory study, we first aimed to identify which kinematic parameters might be controlled by the CNS during repetitive lifting/lowering. Our results suggest that COM kinematic parameters might be of particular interest. Both the GEM-based analysis and SaEn revealed differences between task conditions based on COM kinematics, while neither method yielded important differences for BOX parameters. In the repetitive lifting task examined here, the pick-up and put-down locations of the box were pre-determined, and this constraint may have imposed a secondary goal that affected the BOX trajectory. As such, BOX parameters may not reflect differences due to task asymmetry in terms of relative variability or regularity. This finding is consistent with findings of our earlier study of a prolonged lifting task^36^. In addition, cycle-to-cycle SD, a linear class of methods, captured differences between task asymmetry conditions for the BOX, but not for the COM. Motor noise is often considered as a factor that increases MV, and cycle-to-cycle SD is usually used to capture this sources of MV^44^. The CNS can use kinematic redundancy to overcome such motor noises^45^. That increased cycle-to-cycle SD of variability was found between task conditions only for the BOX implies that the CNS mainly controlled motor noise related to the COM, by manipulating the relative variability and regularity of this kinetic parameter. The results obtained from the three distinct classes of methods suggest that the CNS might primarily control the COM during repetitive lifting/lowering movements. Our findings suggest that identifying a controlled variable (here kinematic parameters) is important, since others have suggested that it is not necessary to determine such variables when using the GEM method^33^.

We also sought in this work to compare metrics of MV between different conditions of task symmetry, which were obtained using different classes of methods. We hypothesized that individuals would use different MV between task conditions, and that the classes of methods would reveal these differences. Our results supported this hypothesis. Both the SaEn and GEM methods provided interesting information about the characteristics of MV in the context of lifting. Specifically, individuals had both less regular movements and higher relative variability (*σ*(*δt*_*T*_)/*σ*(*δt*_*P*_)) in the symmetric lifting condition. As earlier authors have argued^43,46^, these findings suggest that individuals used more constrained patterns in the asymmetric condition, by exerting more control over their COM (see results above for SaEn (*x*_*COM*_) and Fig. 3, bottom). Earlier studies^26,27^ also found that individuals were more stable in an asymmetric condition. Together, the current and earlier results imply that individuals may prioritize flexible patterns over stable ones in a simple task, though this priority can change in a less regular (e.g., asymmetric) task.

In contrast to the SaEn and GEM methods, the linear method (SD) provided information relative to the effects of motor noise on the magnitude of variability of the COM. As discussed above, the increased cycle-to-cycle SD of BOX kinematics with task asymmetry (Fig. 2) might be the result of an increase in motor noise. As such, the observed increased variability of BOX kinematics is undesirable, indicating deviations from a desirable mean operating point. In addition, the overall goal for the participants was to maintain a consistent duration of the lifting/lowering task. More deviations in these kinematic parameters indicate implicitly that the participants were less successful in maintaining task performance. Supporting this, an inverse relationship was evident between overall task performance and metrics obtained using both SaEn and the GEM methods (see Fig. 2-bottom and 3). In other words, task performance deteriorated in parallel with decreasing movement regularity and relative variability from the symmetric to asymmetric conditions. Performance thus appeared to be better in conditions with lower *σ*(*δt*_*P*_). This outcome is consistent with the underlying concepts of the UCM and GEM methods, which posit that variations in the controlled direction affect task performance^4,46^.

Our second hypothesis was also supported, in that the metrics of MV obtained using diverse classes of methods had differing sensitivity to task asymmetry. Effect sizes for cycle-to-cycle SD of the BOX and *T*, SaEn (*x*_*COM*_), and GEM metrics were all high with respect to lifting symmetry. SaEn (*x*_*COM*_) had the highest effect size among all metrics used. While the latter suggests SaEn is the best candidate to explore MV differences, it should be noted that choosing SaEn parameters is challenging^47^ and that different parameters lead to alternative patterns. When we computed SaEn here by selecting the most commonly-used value for the embedding dimension^38^ (i.e., m = 2), we did not find any important differences between task asymmetry conditions. SaEn metrics did differ between the task conditions, however, when the embedding dimension was selected based on the false nearest neighbor approach^48^. GEM-based metrics provided additional results, revealing information about the structure of variability. More specifically, GEM analyses highlighted which parts of variability were beneficial for the examined task (i.e., *σ*(*δt*_*T*_); good variability) and which parts deteriorated performance (i.e., *σ*(*δt*_*P*_); bad variability; Fig. 3 top and middle). As mentioned earlier, the linear method seems useful to evaluate the effects of motor noise and general task performance, given the large observed effect sizes for BOX and T. The Lag-1 autocorrelation method could only predict that movement was corrected more frequently in the non-relevant GEM direction, since *λ*(*δt*_*P*_) was smaller than *λ*(*δt*_*T*_)^39^.

The original studies^27,35^ found that experienced workers may adopt different lifting strategies compared with novices. In the current study, however, our implemented methods did not reveal any differences between these two groups. There are some explanations for the inconsistencies between our results and the previous studies. In the original studies, the authors quantified variability at the joint-angle level. As discussed in the motor control literature, individuals may adopt movement strategies that only change the MV of elemental variables (e.g., joint angles), but without altering MV of controlled variables (e.g., COM). In the study, experienced workers were selected based on a minimal duration of experience in lifting tasks. However, Farrington-Darby and Wilson^49^ suggested that time-on-task alone may not be a sufficient criterion for expertise. As such, the experienced workers in our study could have adopted lifting strategies that altered the variability of their elemental variables (e.g., joint angles), though their lifting experience. However, the duration of experience alone may not have been sufficient for these experienced workers to adopt different MV strategies to regulate (in terms of flexibility and regularity) the controlled variables. Our results are consistent with earlier studies in which low levels of pain only affected inter-segmental coordination pattern^50^, but without changing the overall relative variability^25^. An alternative explanation for the inconsistency between our results and previous studies may be due to sample size. Specifically, the sample size may not have been sufficient to provide a reasonable representation of the behavior of an experienced or novice group^51^. As such, it is unclear whether the results of the previous studies regarding potential differences between the experienced workers and novices were reasonable or a false positive. Future work is need to explore these critical points.

A limitation of this study is that the lifting task was somewhat artificial and constrained (e.g., foot placement was fixed), included only two symmetry conditions, and had a relatively small sample size. As such, we do not know whether or to what extent these results will generalize to other tasks. Regarding the nonlinear class of method, the algorithm employed for determining delay time was not mathematically validated, and it is possible that the number of lifting/lowering cycles used here was insufficient to obtain reliable GEM-based measures. Filtering of raw time series can be also problematic. As Samani *et al*.^38^ discussed, there is a debate regarding the need for or impact of low-pass filtering of raw time series prior to implementing the nonlinear class of methods. For example, England and Granata^26^ recommend filtering, since they believed that high-frequency variations did not reflect musculoskeletal motions. However, other studies have applied the nonlinear class of methods on unfiltered data. As such, future work is needed to address this critical point. It is worth noting that the current study provides results regarding the utility of different methods for quantifying MV in the context of a repetitive lifting task. Future studies should be conducted to evaluate the sensitivity of such methods to diverse individual differences and other task conditions. Finally, we only selected one approach from each class of methods, and examined two kinematic parameters. Future work should explore how other methods and kinematic parameters can reveal more information regarding MV in the context of lifting tasks.

In the context of repetitive lifting tasks, we conclude that the CNS mainly controls the whole-body COM, and that characterizing MV with this kinematic parameter can provide useful information regarding movement regularity and flexibility. Our results indicate that metrics of MV derived using different classes of methods provide complimentary information. The linear class of methods provided information regarding motor noise, while non-linear (SaEn) and equifinality (GEM) methods revealed how the CNS regulates MV to overcome this unwanted motor noise. Our results, together with earlier findings, suggest that the CNS may adopt more stable and less flexible lifting patterns with increased task difficulty and motor noise. It also appears that movement flexibility has an inverse relationship with overall task performance. While both the SaEn and GEM methods are appropriate methods to quantify MV in the context of lifting movements, the latter provided additional information regarding the structures of the “bad” and “good” components of variability.

## Supplementary information

Appendices

## Data Availability

The datasets generated during and/or analyzed during the current study are available from the corresponding author on reasonable request.
