# Supplementary material for: Exploration of different classes of metrics to characterize motor variability during repetitive symmetric and asymmetric lifting tasks
Source: Sci Rep. 2019 Jul 8;9:9821. doi: 10.1038/s41598-019-46297-3 (PMC6614496; doi:10.1038/s41598-019-46297-3)
Supplement: Supplementary file 1 — Appendices [file 41598_2019_46297_MOESM1_ESM.docx]

**Exploration of different classes of metrics to characterize motor variability during repetitive symmetric and asymmetric lifting tasks**

Alireza Sedighi^1^, and Maury A. Nussbaum^2^*

^1^ Bone and Joint Center, Department of Orthopaedic Surgery, Henry Ford Hospital, Detroit, Michigan, 48202 USA

^2^ Industrial and Systems Engineering, Virginia Tech, Blacksburg, 24061 USA

**Appendix A**

The first step in applying the nonlinear class of methods is to reconstruct the state space ^1,2^. This space can be expressed as:

| $X\left( i \right)=[x\left( i \right),x\left( i+\tau\right),\ldots,x(i+\left( m-1 \right)\tau)]$ | (A.1) |
| --- | --- |

where $X(i)$ is the state vector, $x(i)$ is the normalized increment of $x_{COM}$ or $x_{BOX}$, $\tau$ is a delay time, $m$ is an embedding dimension, and $i=1,2,\ldots, N-m\tau$.

Richman and Moorman ^3^ introduced SaEn as a modification of approximate entropy. In their method, self-similar patterns are excluded, and Govindan et al. ^4^ modified the method by including time delay. Based on these approaches, SaEn was calculated as:

| $SaEn\left( x,m,\tau,r \right)=-ln(\Phi^{m+1}/\Phi^{m})$ | (A.2) |
| --- | --- |

To obtain $\Phi^{m}$, we first calculated $C_{i}^{m}(r)$ by counting the number of $j$, such that $d[X(i),X(j)]<r$ and $\left| j-i \right|>\tau$, and where $d[X(i),X(j)]$ is the Chebyshev distance. We used $\left| j-i \right|>\tau$ to remove the temporal correlation effect ^4,5^. Next, we computed the mean of $C_{i}^{m}(r)$. We also calculated SaEn for a wide range of $r$ values (0.05, 0.1, 0.15, 0.2, 0.25, and 0.3). Our results indicated that patterns of SaEn values were consistent for $r_{COM}$ $>$ 0.05 and $r_{BOX}$ $>$ 0.1. As such, we only report SaEn for the most common-used value (i.e., $r=0.2$) ^6,7^. Time delay and embedding dimension were respectively calculated using autocorrelation ^8^ and the false nearest neighbors (FNN) approach ^9^. The calculated mean (SD) values of the time delays were: $\tau_{COM,SYM}=$ 40.92 (3.29), $\tau_{COM,ASYM}=$ 46.5 (4.34), $\tau_{BOX,SYM}$ = 47.52 (2.15), and $\tau_{BOX,ASYM}=$ 49.17(2.59). The embedding dimensions for the symmetric and asymmetric conditions were 5 and 6, respectively. Note that we computed SaEn of the increment data, instead of using the original time series, due to high correlations within these time series ^10^.

**Appendix B**

In a time-based GEM method, any combination of path ($X(i)$) and speed ($V(i)$) in the $i^{th}$ cycle can satisfy the following goal function ^11,12^:

| $f(X\left( i \right),V\left( i \right)=\frac{X(i)}{V(i)}-T$ | (B.1) |
| --- | --- |

In equation (B.1), $T$ is the duration between two consecutive initiation cycles, and it is relatively constant on average. $X(i)$ can be several kinematic values (here, the path of the BOX and COM), and $V(i)$ is the mean speed between two lifting/lowering events. If $X$ and $V$ vary in ways that do not change $T$, then their variations (i.e., ${\delta t}_{T}$) are aligned with the goal. Variations in the lifting parameters, which have effects on the goal (i.e., ${\delta t}_{P}$), are not desirable. Dingwell et al. ^11^ developed a method to calculate these variations, specifically variations in the GEM direction (${\delta t}_{T}$) and non-GEM direction (${\delta t}_{P}$)) for each cycle. In the first step, each variable was normalized to its SD: $X_{n}=X(i)/SD(X(i))$ and $V_{n}=V(i)/SD(V(i))$. By respectively substituting $X(i)$ and $V(i)$ into equation (B.1) with $SD(X(i))X_{n}$ and $SD(V(i))V_{n}$, the rescaled GEM is computed as:

| $\frac{X_{n}}{V_{n}}=\left[ \frac{SD\left( V\left( i \right) \right)}{SD\left( X\left( i \right) \right)}.T \right]=T_{GEM}$ | (B.2) |
| --- | --- |

Normalizing these variables enabled us to compare MV both within and between subjects ^13^. It was assumed that the preferred operation point $(V^{*}, X^{*})$ was the mean of $(V_{n},T_{GEM} \cdot V_{n})$ and that two main variables (i.e., $V_{n}$ and $X_{n}$) are fluctuating around this point. We can calculate these variations as follows:

| $\begin{matrix} {\Delta V}_{n}=V_{n}-V^{*} \\ \Delta X_{n}=X_{n}-X^{*} \end{matrix}$ | (B.3) |
| --- | --- |

Subsequently, equation B.3 is linearized around $(V^{*}, X^{*})$ to compute ${\delta t}_{T}$ and ${\delta t}_{P}$ (for additional details, please refer to Dingwell et al. ^11^).

**References**

1 Abarbanel, H. D., Brown, R., Sidorowich, J. J. & Tsimring, L. S. The analysis of observed chaotic data in physical systems. *Reviews of modern physics* **65**, 1331-1392 (1993).

2 Kugiumtzis, D. State space reconstruction parameters in the analysis of chaotic time series—the role of the time window length. *Physica D: Nonlinear Phenomena* **95**, 13-28 (1996).

3 Richman, J. S. & Moorman, J. R. Physiological time-series analysis using approximate entropy and sample entropy. *American Journal of Physiology-Heart and Circulatory Physiology* **278**, H2039-H2049 (2000).

4 Govindan, R. B., Wilson, J. D., Eswaran, H., Lowery, C. L. & Preißl, H. Revisiting sample entropy analysis. *Physica A: Statistical Mechanics and its Applications* **376**, 158-164, doi:<https://doi.org/10.1016/j.physa.2006.10.077> (2007).

5 Xie, H.-B., Guo, J.-Y. & Zheng, Y.-P. Using the modified sample entropy to detect determinism. *Physics Letters A* **374**, 3926-3931, doi:<https://doi.org/10.1016/j.physleta.2010.07.058> (2010).

6 Zhang, X. & Zhou, P. Sample entropy analysis of surface EMG for improved muscle activity onset detection against spurious background spikes. *Journal of Electromyography and Kinesiology* **22**, 901-907 (2012).

7 Kaffashi, F., Foglyano, R., Wilson, C. G. & Loparo, K. A. The effect of time delay on Approximate & Sample Entropy calculations. *Physica D: Nonlinear Phenomena* **237**, 3069-3074, doi:<https://doi.org/10.1016/j.physd.2008.06.005> (2008).

8 Rosenstein, M. T., Collins, J. J. & De Luca, C. J. A practical method for calculating largest Lyapunov exponents from small data sets. *Physica D: Nonlinear Phenomena* **65**, 117-134 (1993).

9 Kennel, M. B., Brown, R. & Abarbanel, H. D. Determining embedding dimension for phase-space reconstruction using a geometrical construction. *Physical review A* **45**, 3403-3411 (1992).

10 Ramdani, S., Seigle, B., Lagarde, J., Bouchara, F. & Bernard, P. L. On the use of sample entropy to analyze human postural sway data. *Medical engineering & physics* **31**, 1023-1031 (2009).

11 Dingwell, J. B., Smallwood, R. F. & Cusumano, J. P. Trial-to-trial dynamics and learning in a generalized, redundant reaching task. *Journal of neurophysiology* **109**, 225-237 (2013).

12 Cusumano, J. P. & Cesari, P. Body-goal variability mapping in an aiming task. *Biological cybernetics* **94**, 367-379 (2006).

13 Dingwell, J. B. & Cusumano, J. P. Do humans optimally exploit redundancy to control step variability in walking? *PLoS computational biology* **6**, e1000856 (2010).
